# A Grid-Based Framework for Collective Perception in Autonomous Vehicles

**DOI:** 10.3390/s21030744

**Published:** 2021-01-22

**Authors:** Jorge Godoy, Víctor Jiménez, Antonio Artuñedo, Jorge Villagra

**Affiliations:** Centre for Automation and Robotics (CSIC-UPM), Ctra. M300 Campo Real, Km 0.200, Arganda del Rey, 28500 Madrid, Spain; victor.jimenez@csic.es (V.J.); antonio.artunedo@csic.es (A.A.); jorge.villagra@csic.es (J.V.)

**Keywords:** autonomous driving, connected vehicles, cooperative perception, collective perception service, V2X, occupancy grid

## Abstract

Today, perception solutions for Automated Vehicles rely on sensors on board the vehicle, which are limited by the line of sight and occlusions caused by any other elements on the road. As an alternative, Vehicle-to-Everything (V2X) communications allow vehicles to cooperate and enhance their perception capabilities. Besides announcing its own presence and intentions, services such as Collective Perception (CPS) aim to share information about perceived objects as a high-level description. This work proposes a perception framework for fusing information from on-board sensors and data received via CPS messages (CPM). To that end, the environment is modeled using an occupancy grid where occupied, and free and uncertain space is considered. For each sensor, including V2X, independent grids are calculated from sensor measurements and uncertainties and then fused in terms of both occupancy and confidence. Moreover, the implementation of a Particle Filter allows the evolution of cell occupancy from one step to the next, allowing for object tracking. The proposed framework was validated on a set of experiments using real vehicles and infrastructure sensors for sensing static and dynamic objects. Results showed a good performance even under important uncertainties and delays, hence validating the viability of the proposed framework for Collective Perception.

## 1. Introduction

As Advance Driving Assistance Systems (ADAS) evolve, more sensor technologies are embedded within commercial vehicles for environment perception. Today’s most common solutions found in the market, such as Lane Keeping Assist (LKA) or Adaptive Cruise Control (ACC), usually rely on a camera or radar sensor which perceives the area in front of the vehicle. Each type of sensor (e.g., LiDARs, cameras, radars, and ultrasounds) have different perception capabilities, such as range and field of view, and therefore different applications. Moreover, weather conditions do not affect all technologies in the same manner, e.g., radars have a better performance than cameras when facing rain or fog, and cameras are much more sensitive to lighting conditions. Thus, manufacturers often combine several sensors to generate a reliable model of the environment and extract the most relevant features for ADAS.

Regardless of the technology used, on-board sensors are limited by the line of sight of the ego-vehicle, being susceptible to suffer occlusions caused by any other vehicles or elements present on the road. A recent alternative to cope with this problem is found in Vehicle-to-Everything (V2X) communications, which includes both Vehicle-to-Vehicle (V2V) and Vehicle-to-Infrastructure (V2I). In general, communications links have a higher range than on-board sensors, being also more reliable when facing adverse weather conditions.

Thanks to V2X communication, connected vehicles may announce its own presence and intentions to other connected vehicles in the nearby area, expanding thus the ego-vehicle field of view. To make this vision a reality, the European Telecommunications Standards Institute (ETSI) has been working during the last 15 years on different standards for Intelligent Transportation Systems (ITS) applications, where one of the most representative successful examples is the Cooperative Awareness Message (CAM). This message standardizes the dissemination of the ego state, including information such as position, speed, heading or vehicle type.

Presently, the concept of cooperative perception has further evolved, aiming to share not only the own state but also the objects perceived. To that end, different message formats have been proposed for sharing information about perceived objects. In [1] the Cooperative Sensing Message (CSM) was proposed for sharing a description of up to 16 moving objects detected by the ego-vehicle. In [2] the Environmental Perception Message (EPM) was proposed for describing static and moving objects, besides adding information about the ego-vehicle and the sensors on board. Meanwhile, ETSI is working in the standardization of the Collective Perception Message (CPM) [3], which in its current version considers not only information about objects and sensors but also about the free space.

This work focuses on the integration of CPM information for perceiving the environment of an automated vehicle. The ultimate goal is to provide a framework for fusing on-board sensors and external data sources, realizing a high-level description of the environment that considers both objects and navigable space. To that end, the environment is modeled using Dynamic Occupancy Grids (DOG) where occupied, and free and uncertain space is considered. This is done to consider not only sensors measurements but also their associated uncertainty. The use of DOGs minimizes the well-known problem of data association and facilitates the integration of the different types of data received over V2X. Furthermore, the proposed frameworks take into account the dynamic behavior of objects, predicting cell evolution from one step to the next. Besides improving the occupancy estimation, this translates in a better object tracking and a higher resilience to uncertainties and asynchrony in CPM data, which may be critical for subsequent autonomous driving processes, such as motion planning.

The main contributions of this work are therefore the design and analysis of DOG for collective perception and its successful evaluation in a real-world setting with an automated vehicle.

The remainder of this paper is structured as follows. Section 2 provides an overview of related works regarding cooperative perception for Connected Autonomous Vehicles (CAVs). Section 3 details the perception framework used for on-board sensors, based on dynamic occupancy grids. In Section 4, the communications system is described, detailing CPM content and its generation rules. Introduction of CPM information into the occupancy grid is described in Section 5. Section 6 presents the analysis of the performed experiments using real-time data and a CAV prototype. Finally, Section 7 summarizes the works and provides an outlook to future works.

## 2. Related Work

The benefits of using information perceived outside the vehicle and communicated through safety-critical V2X communications has been deeply investigated for more than 20 years, ranging from Cooperative Adaptive Cruise Control [4] to cooperative localization [5], intersection management [6], or even sharing local maps [7]. As a result, an important ADAS-oriented standardization activity has been conducted around Dedicated Short Range Communication (DSRC) technology based on the IEEE 802.11p standard, for which the 5.9 GHz band has been allocated. Some consolidated examples of this trend are the Cooperative Awareness Messages [8], which conveys critical vehicle state information, or the Decentralized Environmental Notification Messages (DENM) [9], which disseminates event-driven safety information by and to the road participants.

The main drawback of these applications is the well-known network effect, where a critical mass of users is required to have a measurable advantage of using the technology, the so-called network effect [10]. To mitigate this limitation the idea of sharing information gathered about objects perceived by the local perception sensors of other connected vehicles is gaining momentum.

Under this paradigm, non-communicating vehicles can be also recognized by V2X-connected vehicles, even when they are not in the line of sight of the ego-vehicle’s local perception system. Thus, an increase in situational awareness is obtained without substantial additional costs. Additionally, several works have already shown, under simulations frameworks, that this cooperative sensing approach improves traffic flow and safety [11].

Different approaches (e.g., [12,13]) for cooperative perception have already been investigated, as information about the perceived objects can be exchanged at track level, at a feature-level or even at a lower-level (occupancy grid or even point clouds). One of the main conclusions of these works is that the communication delay increases with the amount of data transmitted, which suggests avoiding the transmission of unnecessary data. To minimize the bandwidth required for collective perception and reduce the latency, ref. [14] investigated the concept of sharing detected object data instead of raw sensor data. In [2], the message concept was extended with different containers to specify the detected object parameters, sensor configurations and the characteristics of the transmitting vehicle. The resulting message was named Collective Perception Message (CPM), which initially was differentiated [14] following its generating entity (vCPM for vehicles; iCPM for infrastructure equipment). Alternatively, the Environmental Perception Message (EPM) [15] was proposed for exchanging locally perceived object data at feature-level, also including information about the ego state and the Field of View (FOV) of the on-board sensors. The Cooperative Sensing Message (CSM) [1] introduced a hybrid approach for exchanging locally perceived objects at feature and track level.

CPM is gaining interest for several reasons: (i) it is raw sensor data agnostic and therefore vendor-neutral; (ii) it broadcasts only tracked objects and therefore improves communication load; (iii) the representation of multiple perceived objects in the sender body frame to the global sender state is less memory consuming than sharing features to representing thereafter the perceived objects in global coordinates.

The spatial and temporal alignment of incoming data is a crucial aspect to be solved for the massive deployment of cooperative perception systems. Indeed, as a vehicle receives information about dynamic objects from other equipped vehicles or roadside stations, temporal alignment is done by predicting the received object data with different evolution hypothesis (CV, CTRA [16]). Concerning the spatial alignment, one of the key principles is to know the relative pose between data from various sensors of different vehicles, which have been dealt with using relative localization or map merging techniques, considering variants such as landmarks, topological maps, occupancy grid maps, or scan matching [17]. In all these cases, the right management of a large diversity of sensors models and map structures remains an open problem.

Different techniques have been proposed to properly fuse the heterogeneous and decentralized data that is available. A multi-target Kalman Filter is used together with classical association techniques in [18] to manage object-level sharing among vehicles. Iterative Closest Point (ICP) was used in [19] to minimize position and orientation offsets between object lists from different vehicles. Gaussian Mixture Probability Hypothesis Density (GM-PHD) filters were proposed in [20] to track detected cars as rectangular shapes, explicitly considering different measurement models for the different sources of information. Interacting Multiple Model estimators with a sequential multiple hypothesis were tested in [12], using only the position of tracks to make objects associations. As some of these methods only take into account instantaneous spatial information while ignoring the temporal evidence, poor performance can be found in highly dynamic traffic environments. To cope with this problem, ref. [21] combines spatio-temporal evidence and independent vision channel, using Dempster–Shafer theory. Alternatively, ref. [22] proposes a spatio-temporal dissimilarity metric between two reference vehicle states, and presents a greedy algorithm to compute a minimal weighted matching between them. With a completely different approach, ref. [23] showed that collective perception and prediction (P&P) was possible with compressed intermediate representations of a neural network.

The exchanged and fused information needs to be integrated into a representation framework generic enough to incorporate any kind of sensing technology and able to provide the required elements for a safe navigation (free-space representation, perception uncertainty). The use of Dynamic Occupancy Grids, recently explored in a cloud-based realization [24], seems a promising approach to efficiently fuse CPM and on-board sensor information. Although there is several simulation-based studies that analyze the most suitable rules set for CPM dynamic management [25], very few works have investigated in a real-world setting the most convenient in-vehicle data structure for V2X-compliant world modeling. To our knowledge, CPM-oriented DOG suitability is analyzed for the first time in a real automated car.

## 3. Perception Framework

The work presented on this paper has been carried out using the autonomous driving architecture developed by the AUTOPIA Program [26] at the Centre for Automation and Robotics. This section details the core functionality of the perception framework used, while readers may refer to [26,27,28] for an extended description of the architecture modules.

**Figure 1:**
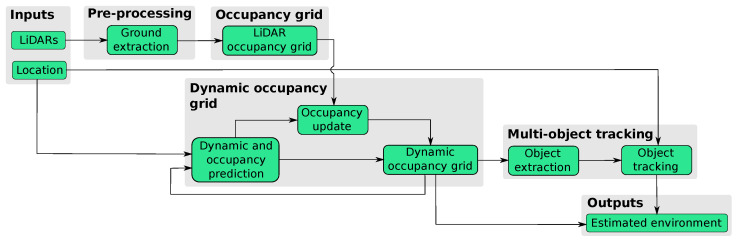
Perception framework scheme.

The perception framework is in charge of modeling the surrounding environment. For this purpose, it takes as input the state (i.e., position, speed and heading) of the ego-vehicle and the data captured by LiDAR sensors. Three LiDARs are used, one 4-layers sensor with 110º FOV, for sensing the front area, and two 16-layers sensors with 360 FOV, for sensing the front-lateral sides of the ego-vehicle. The sensed information, received as independent point clouds, is processed through different stages to obtain a dynamic occupancy grid map and realize an object-level tracking. These stages are grouped into four main tasks: pre-processing, instant occupancy grid, dynamic occupancy grid and multi-object tracking. Figure 1 displays a general view of the framework.

### 3.1. Pre-Processing Task

LiDAR sensors capture a huge amount of data. Therefore, the first task consists of selecting the relevant information. To that end, measurements exceeding a height threshold or impacting the ground are discarded. For the ground extraction the approach presented in [29] is used, since it provides reliable results in real time. This method divides the point cloud into sections and considers them separately. Similar to a polar grid, each section gathers the layers’ beams that share the same angle. Then, the set of points within each section is analyzed taking into account different conditions, such as height difference or unexpected changes in distance.

### 3.2. Instant Occupancy Grid

Once the LiDAR point cloud is filtered, the grid that models the free and occupied space at the current instant of time is computed. The information captured by the three laser sensors is combined following the approach proposed in [30], where a fusion method for dealing with conflicting information is proposed.

First, an independent occupancy grid is computed for each layer of the LiDAR sensors. The sensor’s model estimates the occupancy probability along the cells covered by the beams. This probability is calculated considering that the space between the sensor and the impact is free, the space near the impact is occupied (based on the sensor accuracy) and the space beyond the hit is unknown. In addition to the occupancy probability, a measure of confidence is estimated. The confidence value is calculated taking into account the behavior of the laser sensor, the knowledge of the ground points and the height the beams reach. Thus, the confidence of a beam hitting an obstacle has at that point the maximum value from the sensor to the impact, decreasing toward zero as it goes behind the obstacle. Likewise, the estimation produced by a beam that hits the ground is reliable just until the impact. Lastly, the confidence on free cells of higher layers is decreased. This is done according to the fact that relevant objects with different heights can be found, but the freedom found above a certain threshold does not imply that the space is navigable (e.g., laser beams hit the upper and lower section of a car, but they go through the windows).

As a final step on this task, all the estimated grids are fused according to the Linear Opinion Pool, which combines the layers’ information as a weighted sum [31]. By taking into account the confidence of each measurement, a better estimation is achieved. For example, for a layer that hits the ground at a certain distance, the occupancy probability computed beyond the impact is not reliable at all; therefore, this estimation should not affect the one done by upper layers that may eventually be able to reach further distances.

### 3.3. Dynamic Occupancy Grid

To determine the dynamic state of the grid’s cells, the instant occupancy grid and ego-vehicle pose are used as inputs for updating a dynamic version of the occupancy grid. To that end, the recent history of the cell evolution is taken into account. Multiple approaches can be found in the literature for computing a dynamic occupancy grid. The most promising ones use particles filters to infer the occupancy and dynamic state of the cells. This work uses this approach and is inspired on [32,33,34].

The implementation of this algorithm exceeds the scope of this paper. Thus, a brief introduction to the main flow is presented below. Readers may refer to the cited works for more details.

Considering a set of particles distributed over the grid, the dynamic state of a cell is defined by the dynamic state of the particles within it. Likewise, the occupancy probability at the cell equals the sum of the particles’ weights. These two aspects define the interaction between the grid and the Particle Filter.

In this work the occupancy probability has been substituted by the Dempster–Shafer masses of evidence [35,36], being the frame of discernment the events *occupied* and *free* Ω={O,F}. Thus, the occupancy mass of a cell equals the sum of particles’ weights within it. Please note that when the occupancy probability is used, unknown areas tend to gather multiple particles without providing any benefit. However, the Dempster–Shafer masses of evidence allow the distinction between known and unknown areas, making new particles to spread only over cells reporting evidence for occupancy. This significantly reduces the number of particles needed and, hence, the computational cost.

The dynamic occupancy grid algorithm has three main steps: (i) prediction; (ii) update; (iii) resample and spread of new particles.

At prediction step, it is assumed that the occupancy of a cell can be transferred to other cells depending on its dynamic state. Knowing the relation between particles and cells, this transition is done by particles prediction. Hence, the predicted, or a priori, state of a cell depends on the particles found within it after applying the process model and compensating the ego-vehicle displacement. In turn, the predicted free mass is estimated as in a static grid map, its value is multiplied by a discount factor that models the decreasing reliability of the estimation as time passes. As the total of the evidence masses cannot exceed the value of 1, free mass is limited according to the predicted occupied mass. Moreover, if the sum of predicted occupied mass exceeds the value of 1, the weight of the particles is normalized.

The updated, or a posteriori, grid is calculated by combining the predicted and observed occupancies. Since the occupancy state is represented with masses of evidence, the update is done using the Dempster–Shafer combination rule [37]. The updated value is then translated to the particles, normalizing their weights to equal the a posteriori occupancy mass. Since the current perception framework does not include any sensor able to measure velocities, updates rely on laser beams’ information. Despite not having speed information, the algorithm is able to converge, providing a precise dynamic estimation.

As a final step, particles are resampled, and some new particles are created. The addition of new particles has an important role in identifying new objects. To that end, in each cycle new particles are created in cells reporting evidence of occupancy. For newborn particles, the dynamic state is set randomly, within predefined speed boundaries. In turn, resampling avoids the degeneration of the particles set, discarding those with low probability and strengthening the most probable ones.

### 3.4. Object-Level Tracking

The fourth and last task is to extract high-level objects from the dynamic grid. This information is needed by subsequent processes following the perception, such as maneuver and motion planners, that do not handle information at a cell level. Therefore, a higher-level description that filters and gathers data into potentially relevant objects is required. To that end, objects are described as 2D oriented boxes, including a velocity vector and a classification tag.

To cluster the occupied cells into objects, the DBSCAN algorithm [38] is used. From the clustered cells, a first estimation of the surrounding objects is done, extracting also different characteristics for each one such as: position, velocity, bounding box, etc. However, when no association with previous objects is done, some noisy objects may appear from one step to another. As a result, a Kalman Filter has been implemented for object tracking [39]. To reduce the tracking computational cost, objects are associated from one step to another by the nearest neighbor approach [40].

Tracking objects provides continuity and robustness to perception. Moreover, it allows the classification of the objects into four categories: (i) vehicles, (ii) other dynamic object, (iii) static objects and (iv) noise. For example, objects with fewer detections have lower confidence and are therefore considered to be noise while those that historically have a specific velocity and size are classified as vehicles. Vulnerable Road Users (VRUs), such as pedestrians and cyclists, are classified as other dynamics objects in this work. Nevertheless, the object classification can be extended to other categories as is shown in [41].

## 4. V2X Communications

In this work, CPMs are used for sharing information about obstacles between infrastructure sensors and vehicles. On its current version [3], a CPM is composed by an ITS header, common for all application or facility messages, followed by 5 types of containers:

- a Management Container, providing information about the type and reference position of the station (e.g., vehicle or RSU) disseminating the message.
- a Station Data Container, which inclusion and content depends on whether the message is generated by a RSU or a vehicle.
- Optional Sensor Information Containers, describing the most relevant properties of the sensors associated with the station, such as ids, range, location and type.
- Optional Perceived Object Containers (POC), one per every object perceived by the station. These provide information about the objects, such as id, distance, speed, heading and classification.
- Optional Free-Space Addendum Containers, describing the computed free space detected by sensors.

Each container is composed of a sequence of optional or mandatory fields. As a result, the message structure can be adapted to the available information for each sensor, object and free space within the message; reducing the communication channel load to the strict necessary. In this work CPMs include all containers but the free-space container. Table 1 lists the fields contained in the Perceived Object Container. In addition to mandatory fields, the optional ones marked with * were also included for all objects.

Some fields within the POC, such as distances and speeds, include not only the value of the component but also the confidence associated with it. For each CPM, the inclusion or not of optional containers relies on a set of generation rules defined for each type, aiming to reduce the channel load while focusing on reporting relevant changes in the dynamic environment. Thus, CPMs are transmitted cyclically, with a period that may vary from 100 ms to 1 s depending on the message content.

After an object is first detected, it automatically triggers a CPM event that is sent as soon as the minimum period is satisfied. After that, for the perceived object to be included in the next CPMs at least one of the following conditions must be met since the last time it was included:

- Its absolute position has changed by more than 4 m.
- Its absolute speed has changed by more than 0.5 m/s.
- Its velocity vector has changed its orientation by more than 4 degrees.
- The time elapsed exceeds 1 s.
- The object is classified as a person or animal and the time elapsed exceeds 500 ms.

In case the last rule applies to any of the perceived objects, all those classified as person or animal are included in the next CPM. The individual inclusion of these class of objects is therefore avoided, reducing message generation frequency.

## 5. Enhanced Perception

**Figure 2:**
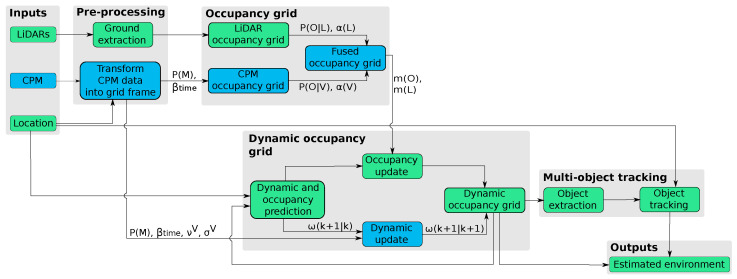
Inclusion of the CPM’s data into the perception framework.

This section describes the inclusion of V2X information into the perception framework presented in Section 3. Figure 2 shows how the CPM information is included into the framework. New steps are colored in blue, denoting the main variables involved in the data fusion. As can be seen on the scheme, perception outputs remain the same: (i) a grid map describing the surrounding in terms of occupancy and dynamic; and (ii) an object-level description of the relevant obstacles found within the grid. Please note that by including data from CPMs the perception range can be extended.

Despite containing high-level description of the objects perceived by external sources, CPM data is fused into the occupancy grid and not into the multi-object tracking directly. This has been done for two reasons: (i) it takes advantage of the grid maps for data fusion and (ii) it allows consideration of a sensor model associated with CPM’s objects uncertainties. As shown in the literature, grid maps are an excellent framework for data fusion [42]. Different types of information from different sources can be equally represented at cell level (e.g., free-space measurements and free-shape objects). Moreover, its low-level representation facilitates the well-known data association problem that is one of the major difficulties in multi-object tracking.

CPMs are available at a variable rate, following the generation rules presented in Section 4, which means on-board sensors are always the primary source of information about the environment. Nevertheless, V2X information is really valuable when it provides data from areas outside the ego-vehicle’s field of view. Therefore, the goal is to fuse the information considering that embedded and external sources are credible and independent enough, being able to model the environment without the need for the other to necessarily exist. Moreover, when having several sources as inputs, the fusion must take into account the reliability of each source.

As already mentioned, CPMs contain information about perceived objects. For each CPM object, its state is defined as:

(1) $$X_{k}^{V,i}=\left\{p_{x},p_{y},\nu_{x},\nu_{y},\theta ,l_{1},l_{2}\right\}$$

where *V* denotes the object received via V2X communications, *i* refers to the *i*th object, *k* is the discrete instant of time, $p_{x}$ and $p_{y}$ are the 2D positions of the object, $\nu_{x}$ and $\nu_{y}$ are its 2D velocities, θ is its heading and $l_{1}$ and $l_{2}$ are the length and the width of the object’s bounding box, respectively.

### 5.1. CPM’s Pre-Processing Step

CPM’s objects are received with a variable rate that it is not synchronized with the grid generation cycle. Furthermore, these objects are referenced to the global frame and their data is associated with some uncertainty values. Therefore, CPM data must be pre-processed before being included into the ego-vehicle’s grid.

To cope with CPM’s asynchrony, objects are stored when received and propagated over a certain period of time, until new object’s data is received, or it is discarded by age. At each grid cycle, the last-known state of the objects is predicted according to the following dynamic model:

(2) $${\hat{X}}^{V,i}\left(t_{c}\right)=\left[\begin{array}{ccccccc} 1, & 0, & \Delta t^{i}, & 0, & 0, & 0, & 0\\ 0, & 1, & 0, & \Delta t^{i}, & 0, & 0, & 0\\ 0, & 0, & 1, & 0, & 0, & 0, & 0\\ 0, & 0, & 0, & 1, & 0, & 0, & 0\\ 0, & 0, & 0, & 0, & 1, & 0, & 0\\ 0, & 0, & 0, & 0, & 0, & 1, & 0\\ 0, & 0, & 0, & 0, & 0, & 0, & 1 \end{array}\right]\cdot X^{V,i}\left(t_{m}^{i}\right)$$

being $t_{c}$ the current time, $t_{m}^{i}$ the time when the measurement of the *i*th object was taken and $\Delta t^{i}$ the time difference between $t_{m}^{i}$ and $t_{c}$.

Please note that the older the last-known state of the object, the greater the prediction error. Thus, the reliability of stored data is degraded over time. For each object, this degradation $\beta_{time}$ is modeled by:

(3) $$\beta_{time}^{i}=1-\frac{\Delta t^{i}}{\Delta t_{max}}$$

where $\Delta t_{max}$ is a design parameter defining the maximum valid object age before it is discarded.

**Figure 3:**
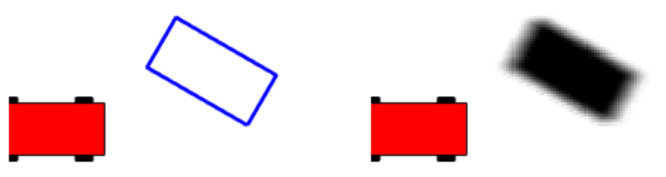
Membership probability map calculated for a CPM object. The ego-vehicle is drawn in red, the CPM object’s box in blue and the membership probability in grayscale.

Once the object state has been predicted, it must be projected into the grid’s cells. To that end, a membership probability $P(M^{c})$ is calculated for each cell, according to the location and shape information of the objects. In an ideal scenario, the cells belonging to an object are those contained inside its bounding box. Therefore, object’s cells would have a membership probability equal to one while for the rest it would be equal to zero. Nevertheless, CPM’s information is associated with uncertainties that must be taken into account. That said, object’s box size is increased proportionally to the object’s box size uncertainties. This method reduces the information loss due to data discretization into cells. Likewise, uncertainty related to object’s location is taken into account following the method proposed in [43]. This is done once per object. Figure 3 shows an example of the membership grid map obtained for an object with uncertainties: $\sigma_{px}=0.5\;m$, $\sigma_{py}=0.1\;m$, and $\sigma_{\theta }=5^{\circ }$.

In case more than one object lay over a cell, the one providing highest probability is chosen. Once assigned, each cell stores the ID, velocity and reliability ($\beta_{time}$) of the object it belongs to. Please note that before storing these values, they must be transformed from the global frame to the local reference system.

### 5.2. CPM Inclusion into the Occupancy Grid

In Section 3.2, the strategy used for fusing the occupancy information from different on-board sensors was explained. One of the main reasons for selecting this approach was its capability of handling situations where there may be information conflicts, because sensors perceive different areas. As the problem tackled in this work falls under that sort of circumstances, the same method is used for including CPM data into the grid. Therefore, the occupancy probability of each cell given the V2X information $P(O^{c}|V)$, is computed. Then both grids, LiDARs’ and CPM’s, are fused applying the Linear Opinion Pool method.

As in this work CPMs include only information about sensors and the perceived objects, with no data about the free space, the model for the occupancy probability is considered to be:

(4) $$P(O^{c}|V)=1$$

This approach could be eventually modified to include information about free space. In that case, the occupancy probability of a cell must be defined by a function that models the degree of occupancy between 0 and 1, being 1 full occupancy and 0 non-occupancy or free space.

Taking into account the reliability degradation associated with CPM measurements, the function of confidence $\alpha^{c}$ is defined as the probability of the cell of belonging to a V2X object weighted by the time elapsed since the measurement was taken:

(5) $$\alpha^{c}\left(V\right)=P\left(M^{c}\right)\cdot \beta_{time}^{i}$$

The Linear Opinion Pool provides the occupancy probability by fusing the information as follows:

(6) $$P\left(O^{c}\right)=\frac{\alpha^{c}\left(L\right)\cdot P\left(O^{c}|L\right)+\alpha^{c}\left(V\right)\cdot P\left(O^{c}|V\right)}{\alpha^{c}\left(L\right)+\alpha^{c}\left(V\right)}$$

As both sources have the same credibility on their own, and by fusing different sources the estimation improves, the confidence of the fused information is calculated as:

(7) $$\alpha^{c}=\min\left(1,\;\alpha^{c}\left(L\right)+\alpha^{c}\left(V\right)\right)$$

Finally, the occupancy probability and confidence values are represented as masses of evidence. As the frame of discernment is the events of *occupied* and *free* Ω={O,F}, cells must have a mass for each one: m(O) and m(F), respectively. These masses are computed as:

(8) $$m\left(O^{c}\right)=\alpha^{c}\cdot P\left(O^{c}\right)$$

(9) $$m\left(L^{c}\right)=\alpha^{c}\cdot \left(1-P\left(O^{c}\right)\right)$$

### 5.3. CPM Inclusion into the Dynamic Occupancy Grid

As previously mentioned, the dynamic state of the cells is estimated using a Particle Filter. However, since the perception framework does not include any sensor capable of measuring objects velocities, particles’ speed are randomly initialized, being the weights updated only by their location. Despite this approach provides good results, its overall performance can be improved by properly initializing the cells and including velocity measurements on weights evaluation. CPM’s information is used for these tasks.

The particles’ state is defined as:

(10) $${\hat{X}}^{p}=\left\{p_{x},p_{y},\nu_{x},\nu_{y}\right\}$$

CPM data is considered to be described by a Gaussian distribution. When a cell belongs to a CPM object, the velocity of the particles that are born inside the object is obtained from these Gaussian distributions. However, the probability of a particle being initialized using the CPM data depends on the membership probability of the cell.

Likewise, particle’s velocity is evaluated using a Gaussian distributed probability density function. Since CPM information was transformed from the global frame to the grid frame, correlations appear between the *X* and *Y* components. Consequently, the likelihood of each particle is evaluated using the bivariate Gaussian distribution $f_{i}\left(\nu_{x}^{p,j},\nu_{y}^{p,j}\right)$:

(11) $$\begin{array}{cc} f_{i}\left(\nu_{x}^{p,j},\nu_{y}^{p,j}\right)=\\ & \frac{1}{2\pi \;\sigma_{\nu_{x}}^{V,i}\sigma_{\nu_{y}}^{V,i}\sqrt{1-{\left(\frac{{\left(\sigma_{\nu_{xy}}^{V,i}\right)}^{2}}{\sigma_{\nu_{x}}^{V,i}\;\sigma_{\nu_{y}}^{V,i}}\right)}^{2}}}\cdot \exp\left(-\frac{1}{2\left(1-{\left(\frac{{\left(\sigma_{\nu_{xy}}^{V,i}\right)}^{2}}{\sigma_{\nu_{x}}^{V,i}\;\sigma_{\nu_{y}}^{V,i}}\right)}^{2}\right)}\cdot \right.\\ & \left.\left[{\left(\frac{\nu_{x}^{p,j}-\nu_{x}^{V,i}}{\sigma_{\nu_{x}}^{V,i}}\right)}^{2}+{\left(\frac{\nu_{y}^{p,j}-\nu_{y}^{V,i}}{\sigma_{\nu_{y}}^{V,i}}\right)}^{2}-2\left(\frac{{\left(\sigma_{\nu_{xy}}^{V,i}\right)}^{2}}{\sigma_{\nu_{x}}^{V,i}\;\sigma_{\nu_{y}}^{V,i}}\right)\left(\frac{\nu_{x}^{p,j}-\nu_{x}^{V,i}}{\sigma_{\nu_{x}}^{V,i}}\right)\left(\frac{\nu_{y}^{p,j}-\nu_{y}^{V,i}}{\sigma_{\nu_{y}}^{V,i}}\right)\right]\right) \end{array}$$

where the superscripts *V* and *p* denote whether the variable refers to a CPM object or a particle, *i* and *j* indicate the object and particle analyzed, and $\nu^{V}$ and $\sigma^{V}$ are the local speed values, mean and standard deviation, for the CPM object.

This evaluation is used to update particles’ weight ω as follows:

(12) $$\omega_{k+1|k+1}^{c,j}=\left(P\left(M_{k}^{c}\right)\cdot \beta_{time}^{i}\cdot f_{i}\left(\nu_{x}^{p,j},\nu_{y}^{p,j}\right)+\left(1-P\left(M_{k}^{c}\right)\cdot \beta_{time}^{i}\right)\right)\cdot \omega_{k+1|k}^{c,j}$$

being *k* the discrete time step and (k+1|k) and (k+1|k+1) refer to the a priori and a posteriori states, respectively. The inclusion of $P(M_{k}^{c})$ models the fact that the particles may or may not correspond to a CPM object.

Please note that after this update, the weights of the particles within the cell must be normalized to respect the relation between particles and occupancy mass explained in Section 3.

### 5.4. Object-Level Tracking

The last task of the perception framework is an object-level tracking that takes as inputs the localization and the environment modeled in the dynamic occupancy grid. As explained at the beginning of this section, all the CPM information is discretized into the grid cells. Therefore, this task remains unchanged.

## 6. Experiments

This section describes a set of experiments conducted to validate the feasibility of the enhanced perception framework under real operating conditions. Experiments were performed in three stages. First, an analysis of the reliability in CPM’s state prediction was done with the goal of determining how CPM uncertainties may affect the grid computation. Then, a test considering only static obstacles was done. In this experiment, the obstacles are fences delimiting a roadworks area. Finally, the last experiment goes a step further and analyses the inclusion of dynamic obstacles.

The latter two experiments were carried out on a segregated environment with an automated vehicle prototype. The vehicle is a conventional Citroën DS3 modified for the automated control of throttle, gearbox, brakes and steering wheel systems. All modifications have been done by the AUTOPIA Program at the Centre for Automation and Robotics in Madrid, Spain. The prototype counts with a set of prioceptive and exteroceptive sensors that are the backbone for location and perception, such as GNSS receiver, LiDARs and stereo camera.

To guarantee the real-time execution of the perception tasks, the framework was implemented using parallel programming with GPUs. In [30,33] several implementation guidelines are provided for the parallel computation of the occupancy grids. The achieved computation time is lower than the cycle of the fastest LiDAR installed on vehicle, which is 80 ms.

**Figure 4:**
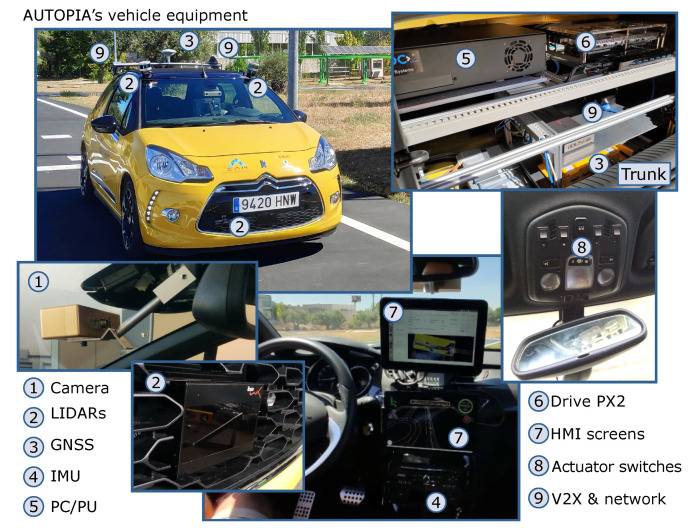
Automated vehicle for experiments.

V2X communications have been implemented using Commsignia RSU & On-Board Units (OBUs). These devices are compatible with IEEE 802.11p and offer a high-level V2X Software Development Kit (SDK) designed to enable automotive suppliers and manufacturers to pilot and validate V2X uses cases. The SDK provides basic services such as Cooperative Awareness Messages and Decentralized Environmental Notification Messages (DENM); and day-one applications such as Forward Collision Warning (FCW) and Lane Change Warning (LCW). Figure 4 shows a general view of the aforementioned components installed on vehicle.

### 6.1. Analysis of Reliability for CPM State Prediction

Section 4 introduced the set of rules defining the generation of CPMs including the Perceived Object Container. Because of these rules, CPMs are generated and sent with a variable period from 100 ms to 1 s, depending on the conditions triggering the transmission. Furthermore, as mentioned in Section 5.1, objects are saved on reception and then, at each grid generation cycle, their state is predicted from the latest observation stored for each one.

The first test was done considering the behavior of a vehicle. This decision was made because static obstacles are not affected by the prediction stage, and for other dynamic objects, such as pedestrians, the maximum CPM gap is limited to 500 ms and therefore its dynamic behavior is too constrained. Hence, prediction error was analyzed considering a theoretical moving passenger car traveling with constant speed and front-wheel angle. To take into account different scenarios, speed was swept from 0 to 25 km/h and the front-wheel angle from 0 to 25 degrees, assuming negative angle values will have a reciprocal output. These values were selected to cover the entire range of CPM generation time under different circumstances.

**Figure 5:**
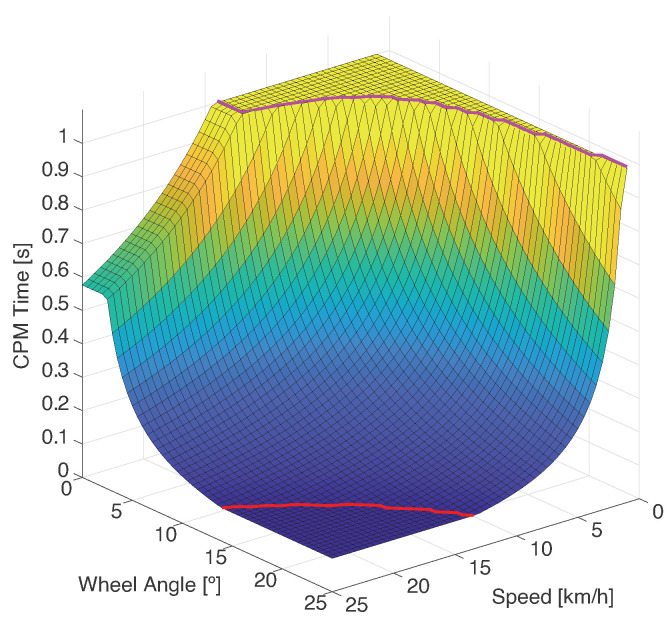
Time elapsed for a CPM event to trigger given different configurations.

Figure 5 shows the variation of the generation time for each combination of speed and front-wheel angle. As can be seen in the graph, the generation time decreases as the speed of the vehicle increases. Likewise, the greater the angle the smaller the generation time. There are two lines on the graph, the magenta one marks when messages start being triggered by the dynamic behavior of the vehicle and the red line marks where the maximum CPM frequency is reached.

**Figure 6:**
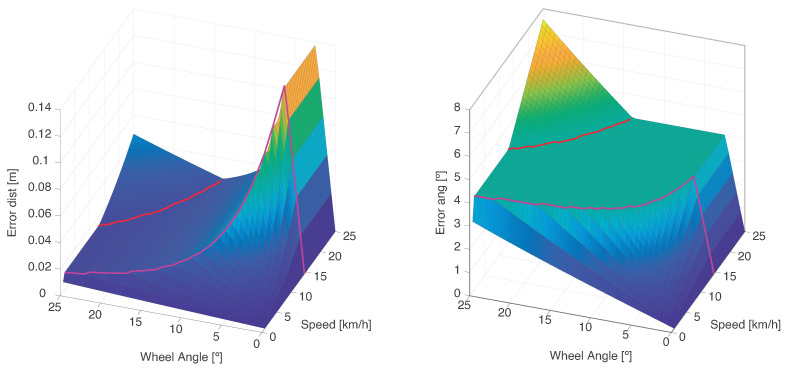
Maximum error achieved when predicting the state until next CPM.

To analyze how the variation in generation time affects the objects prediction, the model defined in Equation (2) was compared with the Constant Turn Rate and Velocity (CTRV) model [44]. The latter better describes the movement of the vehicle for these tests and it is, therefore, taken as ground truth. Figure 6 shows the position and heading difference obtained between the two models. The obtained position error, measured as the Euclidean distance, can be regarded as negligible for all the analyzed cases, remaining always below 15 cm, which is lower than the cell size defined for the perception framework (20 cm). Regarding the heading, a higher error is observed. This is due to the constant heading assumption made by the prediction model. As can be seen on the graph, the flat area indicates that in most of the considered scenarios the CPM message is triggered by the rule of angular displacement. Despite the higher values, the heading error can still be considered valid for the proposed framework. Indeed, as explained in Section 5.2, the orientation of the object is used to compute which cells belong to it. An average vehicle can be covered by a bounding box of 4.5 m length and 2 m width. If the positions of its corners are calculated for 0 and 4 degrees, the disparity obtained is (0.075, 0.16) m. If the disparity is calculated for the maximum obtained angle error, 7.5 degrees, the difference obtained is then (0.15, 0.3) m. Even under these extreme situations, where the vehicle turns without meeting an acceptable comfort criteria, the error obtained would be in the order of magnitude of one to two cells. These error values seem acceptable for ADAS applications, where objects sizes are enlarged with similar values, as explained in Section 5.2.

### 6.2. Real Scenario 1: Roadworks Detection

**Figure 7:**
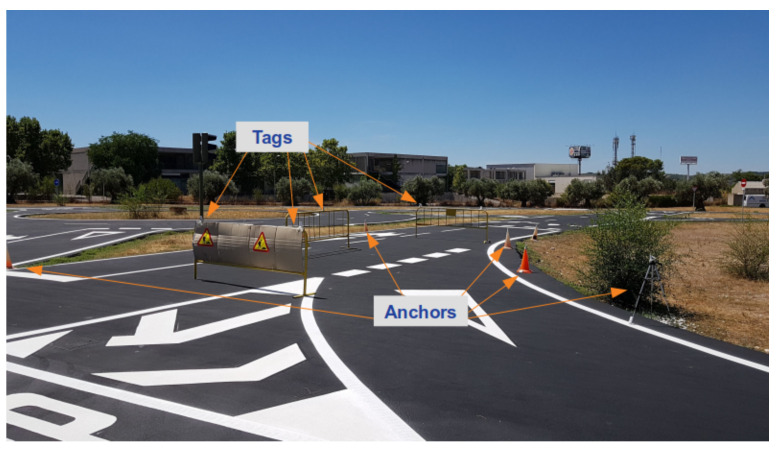
Scenario 1: Roadworks area and UWB sensor.

In this scenario the obstacles are fences, delimiting a roadworks area within the path of the vehicle (Figure 7). The area covered by the roadworks is about 10 m long and 2.5 m wide. Roadworks area is sensed by an Ultra-wideband (UWB) sensor installed on the road infrastructure. Each fence is equipped with UWB tags, which location is determined from the distances to the UWB anchors installed on road by multilateration and transmitted via V2X communications. This scenario shows the main advantages of merging external sources, without which the LiDAR-based perception framework would be unable to model the area behind the first fence.

**Figure 8:**
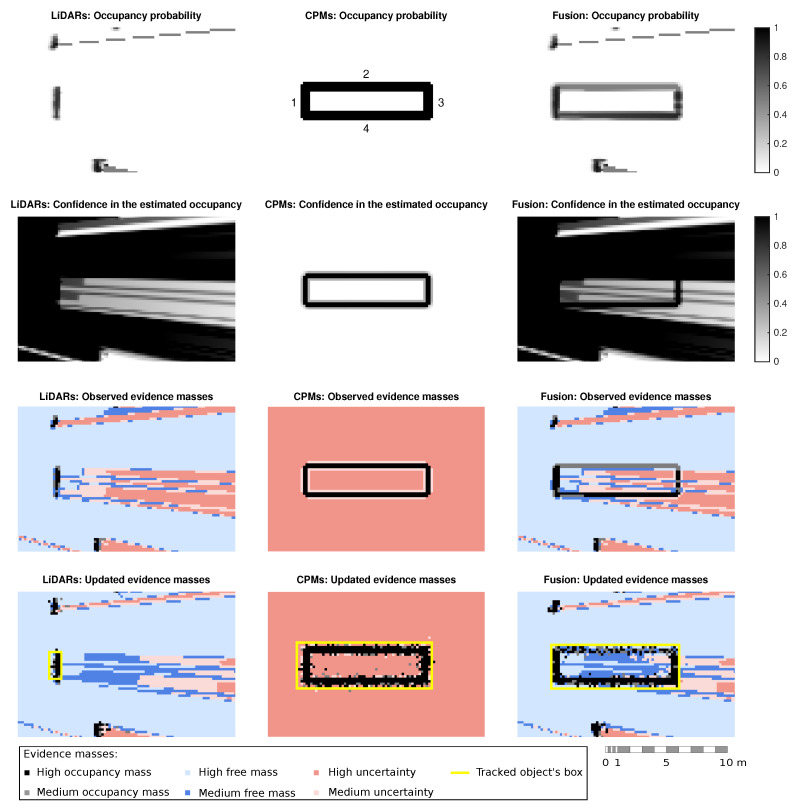
Evolution of the perception algorithm using only LiDAR sensors, only CPM data and the combination of both.

Figure 8 shows a comparison of the occupancy grid calculation using different information sources: (i) LiDAR data only, (ii) CPM data only and, (iii) LiDAR and CPM data. The rows display different steps of the algorithm. The first two rows expose how the information relative to occupancy and confidence is included in the grid. The third row shows the observed occupancy grid represented by evidence masses and the fourth row shows the updated dynamic occupancy grid. To facilitate the reading, fences have been enumerated from 1 to 4, as shown in the second plot of the first row. In all the plots, the ego-vehicle is located at the center of the left side, facing the fences. Since fences are static obstacles, only the fusion of occupancy data is shown. For a better comprehension, evidence masses are shown in the same graph, where cells are colored according to their predominant mass.

As can be seen on the image, LiDARs sensors are unable to sense the area behind the first fence (1) by their own, being the area occluded. Moreover, fences are not as high as vehicles, meaning some reliable laser beams pass over them, which translates as free space. This can be appreciated on the observed masses graphs, where although a barely reliable free space is drawn behind the fence, it ends up converging into a free-space area in the dynamic grid.

On the other hand, when considering CPM data, the limits of the roadworks are detected correctly. Nevertheless, there is no information in the occupancy grid about the drivable space or any other obstacles. The fusion of both sources provides a more realistic representation of the vehicle surroundings. As the third column shows, occupancy modeled from CPM data is correctly merged with LiDAR information, giving a good approximation of both the obstacles and free space. Moreover, the main advantage of applying a weighted fusion is shown at those cells where the longest fences (2 and 4) lay. For the fence 2, both sources have a high confidence in the occupancy estimation, resulting in mean occupancy values. However, at fence 4 the LiDARs’ reliability is nearly zero while the CPM data models occupancy with high reliability, resulting in a high occupancy estimation.

**Figure 9:**
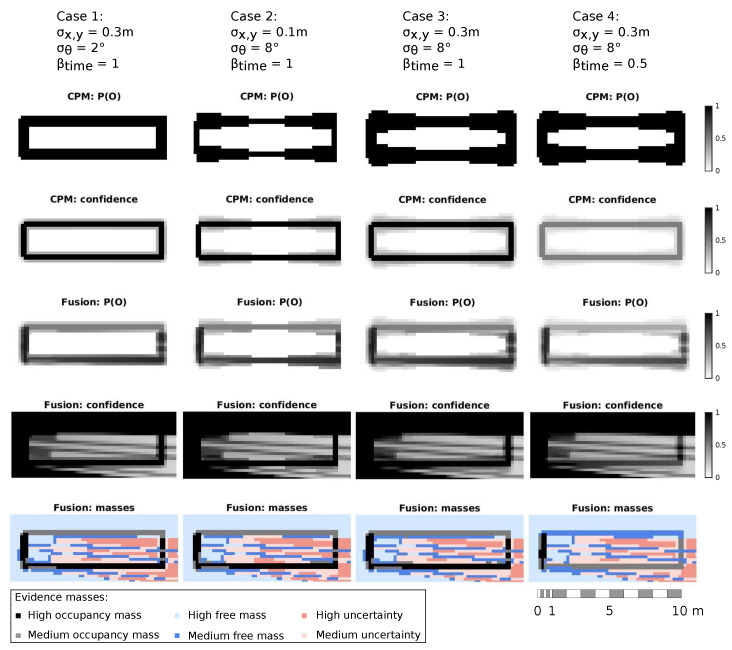
Occupancy grid under different uncertainties.

Figure 9 displays the same scenario but with different uncertainties in the location of the objects. It can be noticed how depending on measurements uncertainty, different cells have chances to be covered by the CPM object, hence modeled with a certain degree of occupancy. For example, if the Intersection over Union (IoU) [45] is calculated between the bounding boxes obtained from the cells covered by the real size of the fences and from the cells covered by the uncertainty propagation, the score obtained decreases as uncertainty increases: IoU(1st case) = 0.834, IoU(2nd case) = 0.722 and IoU(3rd and 4th cases) = 0.625. Additionally, the last column of plots (fourth case) shows the effect of taking into account the time elapsed since the measurements were taken. It can be noticed that if the data of a CPM object is old, its importance during the fusion step is low. Since the barrier 1 is also detected by the LiDAR sensors, it is still estimated with high occupancy. On the other hand, the occupancy estimated for the occluded barriers (2, 3 and 4) has decreased, accordingly to the CPM data reliability. Please note that the values selected for these cases have been deliberately inflated with respect to real uncertainty values for the sake of a better visualization.

Table 2 and Table 3 compare the mean occupancy probability and the mean occupancy mass obtained for 3rd and 4th cases. Since the confidence in the CPM’s estimation decreases as the distance to the object increases, these values are provided for three sets of cells: (i) the cells covered by the CPM’s box, (ii) the cells covered by the uncertainty propagation that are adjacent to the CPM’s box and (iii) the rest of the propagated cells. As can be appreciated, the occupancy of the fence 1 remained high for both occupancy probability and occupancy evidence mass. For the second fence, which is modeled as free by the LiDARs and occupied by the CPM, the occupancy value decreased from 0.5 to 0.33. In the case of the fourth fence, the estimation relied primary on the CPM, therefore the occupancy probability obtained from the fusion is high and hardly decreases with the loss of confidence, while the occupancy mass reflects this fall of reliability reducing its value.

### 6.3. Real Scenario 2: Crossroad with Occlusion

**Figure 10:**
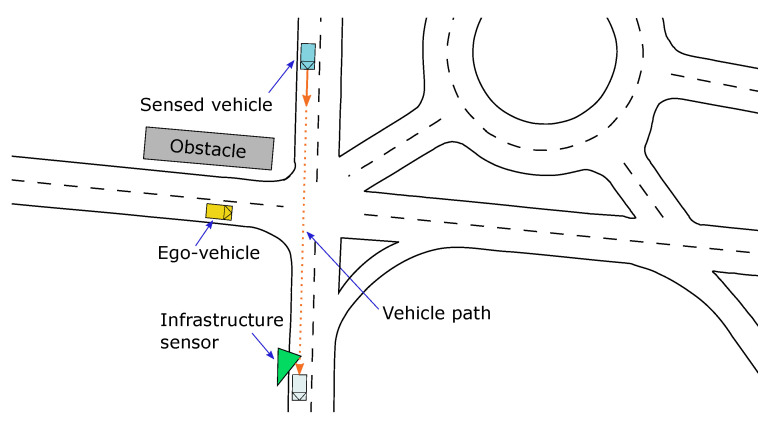
Dynamic obstacles scenario.

For the second experiment, the inclusion of dynamic obstacles is analyzed. In this test, the ego-vehicle is stopped at a crossroad where it must yield to other vehicles. However, due to road occlusion (see Figure 10), it is unable to detect the incoming vehicles using on-board sensors only. At the infrastructure side, a vision-based sensor detects the incoming vehicles and transmits the objects information via V2X communications.

**Figure 11:**
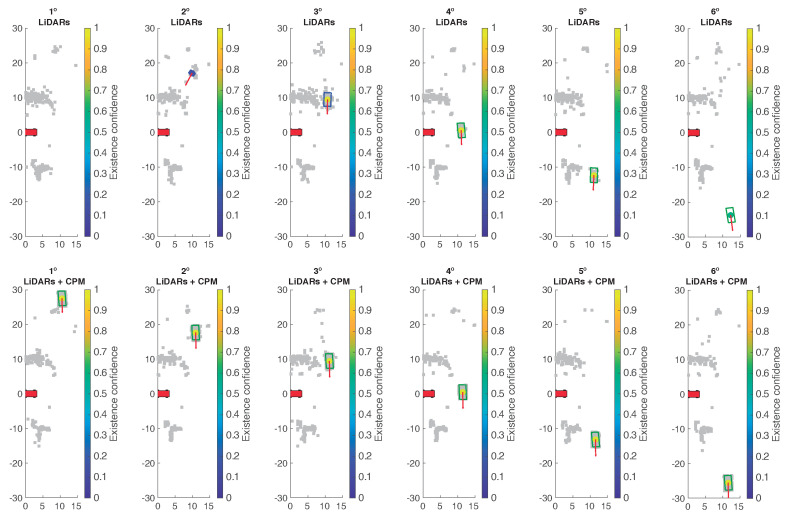
Object tracking with and without CPM data.

Figure 11 shows a comparison between addressing the crossroad without and with CPM data. In all graphs, the occupied cells are represented in grey while the tracked state of the crossing vehicle is represented by the bounding box, its center point and its speed vector. The box is colored according to object classification: green, if classified as vehicle, or blue, if marked as dynamic non-vehicle. Object classification relies on the bounding box’s size and speed value. The color of the center point indicates the confidence value associated with the track, estimated from the number of times the object has been detected. In this experiment the uncertainty related to the object pose has a standard deviation of 10 cm for its location and it is negligible for its orientation. The standard deviation of the velocity longitudinal and lateral components are 0.5 and 1.0 m/s, respectively.

As can be seen on frames evolution, when using only information from on-board sensors the ego-vehicle is unable to track the crossing vehicle correctly. At the first frames, there is a low number of occupied cells, which translates in an unreliable tracking of the moving vehicle. At about 20 m the object is first detected but it is not classified as a vehicle until it is already crossing the intersection. Moreover, its tracked state is quickly lost once it exits the intersection. On the other hand, when CPM data is taken into account, all the occluded areas are covered by the infrastructure sensor. Therefore, a better representation of the occupied space is achieved, which translates in a sooner and more reliable tracking and classification of the moving vehicle.

**Figure 12:**
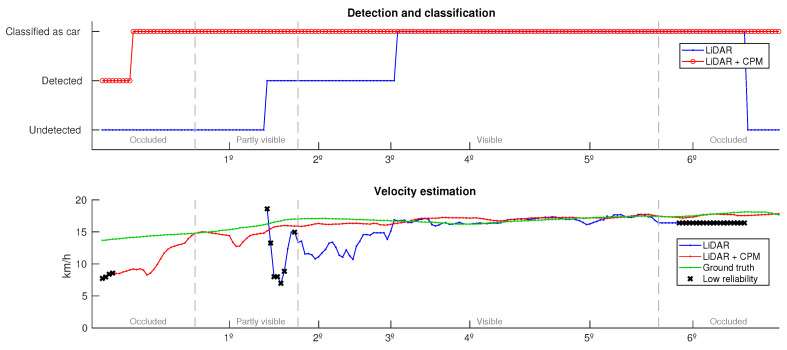
Comparison of the velocity and classification obtained with and without CPM data.

Finally, Figure 12 compares the tracked vehicle evolution in terms of classification and estimated object’s velocity. For a better comprehension of the scene, the frames shown in Figure 11 and the vehicle’s visibility are marked over the x-axis. As can be seen on the top graph, CPM data improves both detection and classification of the object. If only LiDARs are used, the vehicle is tracked for a period of time of 11.6 s, being classified as a vehicle for 8.4 s. On the contrary, when using both sources of information, the vehicle is detected almost all the time it is within the range of the grid map, 19.3 s, and the classification is more quickly achieved, taking only 0.65 s. Moreover, the bottom graph shows that the inclusion of CPM results in a better dynamic estimation, the maximum error achieved without CPM data is 9.7 km/h while if using CPM data is 6.1 km/h, also the mean error is reduced from 1.6 km/h to 1.3 km/h. In the bottom graph, unreliable measurements (existence confidence <0.75) have been denoted with black crosses.

## 7. Conclusions and Future Works

This work proposes a framework for realizing collective perception in autonomous vehicles. The proposed approach uses occupancy grids for fusing data from on-board sensors with CPS messages received via V2X. As main advantage, this method allows consideration of V2X as an additional sensor to the vehicle, helping with the data association problem. Moreover, by taking into account the dynamic behavior of cells, occupancy is evolved from one step to the next, reflecting in a better occupancy estimation and object tracking.

The first experiment analyzed the effect of CPS generation rules in object prediction. The entire range of generation time (100–1000 ms) for CPMs was covered under different conditions by sweeping along the vehicle state variables. Results showed the prediction error to be negligible in all the cases considered, being mostly lower than the cell size defined for the occupancy grid. As expected, CPS integration allowed to enhance vehicle perception. The set of experiments conducted with real vehicles and infrastructure sensors showed the framework capability for sensing static and dynamic objects while coping with object occlusions and messages asynchrony.

From the promising results obtained in this work, future activities will be focused on: (i) analyzing the influence of Collective Perception in other modules related to autonomous driving such as maneuver and motion planners; (ii) introducing a method for adapting the trust on external sensors and avoid/detect third-party attacks such as data tampering; and (iii) full deployment of CPS, transmitting perceived objects to nearby vehicles and analyzing the influence of object redundancy in messages [46]. In addition, the integration of a stereo camera in the test vehicle will allow the exploration of the framework capability to include new data features into the grid and to compare the performance with vision-based algorithms for object detection and classification.

## Figures and Tables

**Table 1:** Perceived Object Container in CPM. Optional fields marked with * were used on this work.

Field Name	Mandatory	Description
objectID	yes	Object unique identifier
sensorIDList	no	Ids of sensors detecting the object
timeOfMeasurement	yes	Time difference from the message’s generation delta time to the time of the measurement of the object
objectAge	no	Age of the described object
objectConfidence	yes	Confidence associated with the object
xDistance	yes	Absolute distances to detected object from the ITS-S’s reference point in xyz directions at the time of measurement
yDistance	yes	
zDistance	no	
xSpeed	yes	Relative speed of the detected object from the ITS-S’s reference point in xyz directions at the time of measurement
ySpeed	yes	
zSpeed	no	
xAcceleration	no	Relative acceleration of the detected object from the ITS-S’s reference point in xyz directions at the time of measurement
yAcceleration	no	
zAcceleration	no	
yawAngle	no *	Relative yaw angle of object from the ITS-S’s reference point
planarObjectDimension1	no *	Dimensions of the object as provided by the sensor or environmental model
planarObjectDimension2	no *	
verticalObjectDimension	no *	
objectRefPoint	yes	Reference point on the perceived object relative to which the measurement data is provided
dynamicStatus	no	Classification of a perceived object towards its capabilities to move
classification	no	Classification of the described object
matchedPosition	no	Map-matched position of an object

**Table 2:** Mean occupancy probability estimated for the fences.

	Fence 1		Fence 2		Fence 3		Fence 4	
	**Case 3**	**Case 4**	**Case 3**	**Case 4**	**Case 3**	**Case 4**	**Case 3**	**Case 4**
**CPM bounding box covered cells**	0.87	0.83	0.50	0.33	0.74	0.59	0.78	0.64
**Neighboring propagated cells**	0.62	0.57	0.20	0.11	0.43	0.28	0.46	0.30
**Distant propagated cells**	(0 cells)	(0 cells)	0.12	0.07	(0 cells)	(0 cells)	0.22	0.13

**Table 3:** Mean occupancy mass estimated for the fences.

	Fence 1		Fence 2		Fence 3		Fence 4	
	**Case 3**	**Case 4**	**Case 3**	**Case 4**	**Case 3**	**Case 4**	**Case 3**	**Case 4**
**CPM bounding box covered cells**	0.87	0.83	0.50	0.33	0.74	0.47	0.78	0.50
**Neighboring propagated cells**	0.62	0.57	0.20	0.11	0.26	0.13	0.26	0.13
**Distant propagated cells**	(0 cells)	(0 cells)	0.10	0.06	(0 cells)	(0 cells)	0.12	0.06

## Data Availability

Not applicable.
